# Attenuated selective attention during visual perspective taking in autism: A combined behavior, ERP, and pupillometry study

**DOI:** 10.1111/jcpp.70156

**Published:** 2026-04-16

**Authors:** Leonie Polzer, Nico Bast, Sara Boxhoorn, Andreas Mühlherr, Hannah Mössinger, Magdalena Schütz, Christine M. Freitag, Christina Luckhardt

**Affiliations:** ^1^ Department of Child and Adolescent Psychiatry, Psychosomatics and Psychotherapy University Hospital Frankfurt, Goethe‐University Frankfurt Frankfurt am Main Germany

**Keywords:** Autism, selective attention, event‐related potential, pupil dilation, perspective taking

## Abstract

**Background:**

Altered social cognition in autism may be influenced by difficulties in domain‐general functions, such as selective attention. Here, we manipulated the requirement for selective attention during a visual perspective taking (VPT) task and used neurophysiological indices to delineate underlying mechanisms.

**Methods:**

43 autistic and 38 neurotypical children and youth completed a VPT paradigm. Participants' and an avatar's perspectives were either congruent or incongruent, manipulating the demand for selective attention to inhibit irrelevant information processing. Group differences in dependence on the attended perspective and congruency were examined for behavioral performance, event‐related potentials (ERPs), and in a subsample, stimulus‐evoked pupil response (SEPR).

**Results:**

Autistic participants showed reduced accuracy when rating an avatar's perspective and pronounced perspective‐related differences in a late‐frontal slow wave (LFSW). Incongruency elicited stronger effects on behavioral performance and LFSW in autistic relative to neurotypical participants. Additionally, generally larger and more sustained SEPR was observed for autistic participants, reflecting an alteration that was dissociable from LFSW differences. Across groups, SEPR and ERP measures explained both distinct and partially overlapping variance in response slowing.

**Conclusions:**

Findings support attenuated VPT abilities and difficulties to deploy selective attention in autistic youth. Altered fronto‐cortical activation patterns in the LFSW likely reflect an increased top‐down involvement in VPT. Concurrently, pupil responses indicate an increased cognitive effort when reporting visual perspectives. Findings emphasize a co‐characterization of social cognition difficulties by social and domain‐general functions in autism.

## Introduction

Autism spectrum disorder (ASD) is associated with atypical social cognition, including difficulties in inferring others' mental states (Velikonja, Fett, & Velthorst, 2019). Among these processes, visual perspective taking (VPT) describes the ability to understand other people's visual perception of the environment. At a basic level (VPT‐1), it requires comprehending whether objects are visible to another person or not, whereas more demanding tasks (VPT‐2) involve mental rotation abilities to understand how visual input looks to others (Flavell, 1977).

During infancy and toddlerhood, atypical early precursors of VPT such as gaze following are frequently linked to autism (Gillespie‐Lynch, Elias, Escudero, Hutman, & Johnson, 2013; Thorup, Nyström, Gredebäck, Bölte, & Falck‐Ytter, 2016). Evidence for explicit VPT performance in autistic toddlers and preschoolers is sparse, although attenuated VPT‐1 performance has been reported (Warreyn, Roeyers, Oelbrandt, & De Groote, 2005). During middle childhood and adolescence, autism is more consistently linked to reduced VPT‐2 performance (Hamilton, Brindley, & Frith, 2009; Ni, Xue, Cai, Wen, & He, 2021) or alternative task‐solving strategies (Conson et al., 2015; Pearson, Marsh, Ropar, & Hamilton, 2016). By adulthood, differences are reported less consistently (David et al., 2010; O'Connor, Plant, & Riggs, 2023), indicating a delayed and qualitatively altered developmental trajectory rather than global difficulties. While VPT differences in autism have been more consistently observed in VPT‐2 paradigms (Pearson, Ropar, & Hamilton, 2013), VPT‐1 tasks offer a methodological advantage for isolating specific cognitive mechanisms by engaging fewer concurrent processes.

Beyond social processing, altered domain‐general functions have been associated with social cognition differences in autism (Bottema‐Beutel, Kim, & Crowley, 2019; Carlson, Mandell, & Williams, 2004). Visual processing depends on selective attention, which describes a differential enhancement of relevant and inhibition of irrelevant stimulus processing (Desimone & Duncan, 1995). During the calculation of visual perspectives, irrelevant input comprises stimuli that are not congruently available to both perspectives and must be selectively disregarded. Perspective incongruency thus increases demands for selective attention. Autistic individuals often exhibit reduced selective attention (Geurts, van den Bergh, & Ruzzano, 2014; Keehn, Nair, Lincoln, Townsend, & Müller, 2016), which may contribute to VPT difficulties.

Perspective congruency during VPT is commonly manipulated using the dot task, in which participants rate the number of targets visible from their own and an avatar's perspective (Samson, Apperly, Braithwaite, Andrews, & Bodley Scott, 2010). Perspectives can be congruent or incongruent due to the occlusion of parts of the scene from the avatar's viewpoint. In neurotypical adults, perspective incongruency impairs reports of the own perspective, and more strongly, ratings of the avatar's perspective (Samson et al., 2010), highlighting increased incongruency‐related demands on selective attention.

Behavioral findings from dot‐task studies in autistic adults are heterogeneous. Two studies reported opposing perspective effects, with faster responses for the own compared to the avatar's perspective either only in neurotypical participants (Tei et al., 2019) or only in autistic participants (Schwarzkopf, Schilbach, Vogeley, & Timmermans, 2014). Both studies found incongruency‐related slowing during avatar‐perspective judgments across groups, whereas effects on the own perspective were reported in only one study (Schwarzkopf et al., 2014). A third study further supported selective incongruency effects during avatar‐perspective judgments, which could be observed in both groups when participants needed to switch between rating the own and the avatar's perspectives, but were limited to autistic participants when perspectives remained constant across trials (Doi, Kanai, Tsumura, Shinohara, & Kato, 2020). Given developmental trajectories, clearer patterns may emerge during childhood and adolescence.

Expanding behavioral evidence to neurophysiological signatures could further delineate mechanisms underlying VPT alterations. Social cognition processing recruits temporo‐parietal and prefrontal regions (van Overwalle, 2009), which show diverging activation during VPT in autism (Eack, Wojtalik, Keshavan, & Minshew, 2017; Seymour, Rippon, Gooding‐Williams, Wang, & Kessler, 2025). Event‐related potentials (ERPs) provide time‐resolved indices of social cognition processes (Amodio, Bartholow, & Ito, 2014) and can reveal processing alterations not evident at the behavioral level (Karhson & Golob, 2016; Lartseva, Dijkstra, Kan, & Buitelaar, 2014). In neurotypical adults, parieto‐occipital P200, central‐parietal P3, and late‐frontal slow‐wave (LFSW) components show interaction effects of perspective and congruency in the dot task, alongside specific congruency effects on P200, P3, and LFSW amplitudes, and perspective effects on P3 latency (Ferguson, Brunsdon, & Bradford, 2018; McCleery, Surtees, Graham, Richards, & Apperly, 2011; Peng et al., 2018; Sæther et al., 2021). Accordingly, ERPs have been proposed to delineate self‐other distinctions (P3), top‐down processing during response selection (LFSW), and earlier attentional processes (P200) in the dot task.

Differences in processing may further be informed by indices of cognitive effort. Norepinephrine releases from the locus coeruleus (LC) relate to an increased activation of the sympathetic nervous system (Samuels & Szabadi, 2008), which is indexed by the stimulus‐evoked pupil response (SEPR; Joshi, Li, Kalwani, & Gold, 2016; Megemont, McBurney‐Lin, & Yang, 2022). During cognitive task engagement, SEPR increases as a function of cognitive effort (van der Wel & van Steenbergen, 2018), providing an index of effort allocation that may differ during VPT in autism.

In this study, we investigate VPT and its underlying mechanisms in autistic and neurotypical youth, using established ERPs (P200, P3, LFSW) and SEPR, with selective attention demands manipulated via perspective incongruency. Given heterogeneous prior findings, we examine group differences in behavioral VPT performance without a priori hypotheses. We expect altered neurophysiological VPT processing in autism, reflected in ERP or SEPR signatures, and stronger effects of perspective incongruency in autistic individuals. Finally, we assess whether the impact of incongruency on VPT performance differs between groups and explore links between neurophysiological markers and behavior.

## Methods

### Sample

Participants were 43 autistic (ASD) and 38 neurotypical (NT) individuals aged 10–17 years, who were recruited from a volunteer database. Groups were matched for IQ using full matching and did not differ in age and sex (see Table 1). Pupillometry was assessed in a subsample (ASD: *n* = 28, NT: *n* = 27). Inclusion required an IQ ≥ 70 based on standardized composite scores from verbal comprehension and perceptual organization subscales of the German WISC‐III/HAWIK‐III (Tewes, Schallberger, & Rossmann, 1999) or WAIS‐III/HAWIE‐III (von Aster, Neubauer, & Horn, 2006). Inclusion of autistic participants required an expert ASD diagnosis according to ICD‐10 using the German versions of the ADOS/ADOS‐2 (Poustka et al., 2015; Rühl, Bölte, Feineis‐Matthews, & Poustka, 2004) and the ADI‐R (Bölte, Rühl, Schmötzer, & Poustka, 2006). If ADI‐R values were unavailable, inclusion required an ADOS classification of ‘autism’ (*n* = 2). Additional exclusion criteria are described in detail elsewhere (Boxhoorn et al., 2022). Seven autistic participants met ICD‐10 criteria for ADHD based on parent report (FBB‐ADHS; Döpfner, Görtz‐Dorten, & Lehmkuhl, 2008). Neurotypical participants were excluded if CBCL/4‐18R Total Scores (Achenbach, 1991) were within the clinical range (*T* > 60). Primary caregivers and participants gave their written informed consent prior to study participation. The project was approved by the ethics committee of the Medical Faculty at the University Hospital Frankfurt (votum 416/17). Data from overlapping samples have previously been published (Boxhoorn et al., 2022; Schütz et al., 2023).

**Table 1:** Sample description

Full sample	*M* [*SD*]	*M* [*SD*]	*p*‐Value
	ASD (*n* = 43)	NT (*n* = 38)	
Sex (*n* male/*n* female)	39/4	28/10	.084
Age (years)	14.41 [2.24]	15.13 [1.84]	.115
IQ	101 [12.68]	106.18 [9.45]	.067
Handedness (LQ)	58.61 [46.43]	74.60 [40.86]	.103
CBCL internalizing (T‐score)	63.02 [8.43]	46.74 [7.88]	<.001
CBCL externalizing (T‐score)	57.16 [11.91]	42.94 [6.44]	<.001
CBCL total (T‐score)	63.05 [9.77]	43.60 [7.15]	<.001
SRS‐16 (T‐score)	61.29 [3.99]	48.43 [4.37]	<.001
ADOS CSS	6.36 [1.75]	–	–
ADI‐R social interaction	15.51 [4.81]	–	–
ADI‐R communication	12.24 [4.95]	–	–
ADI‐R rituals	4.56 [2.27]	–	–
**Pupillometry subsample**	**ASD (*n* = 28)**	**NT (*n* = 27)**	
Sex (*n* male/*n* female)	24/4	19/8	.293
Age (years)	13.71 [2.18]	15.55 [1.84]	.001
IQ	102.36 [14.19]	105.52 [9.42]	.334
Handedness (LQ)	53.67 [48.93]	82.61 [19.63]	.006
CBCL internalizing (T‐score)	60.82 [7.54]	45.96 [7.20]	<.001
CBCL externalizing (T‐score)	56.14 [12.05]	42.65 [6.44]	<.001
CBCL total (T‐score)	61.50 [9.52]	43.62 [6.42]	<.001
SRS‐16 (T‐score)	61.62 [3.61]	47.84 [3.40]	<.001
ADOS CSS	6.36 [1.70]	–	–
ADI‐R social interaction	14.96 [5.27]	–	–
ADI‐R communication	11.12 [4.92]	–	–
ADI‐R rituals	4.38 [2.14]	–	–
In‐trial SEPR data availability (%)	89.67 [12.97]	91.53 [6.45]	.502

Values are presented as mean (*SD*), unless otherwise specified. *p*‐values are derived from two‐sided unpaired *t*‐tests; only sex differences were assessed using Pearson's Chi‐squared test. ADOS CSS could not be calculated for one ASD participant, as their age exceeded the standardized range provided in the manual. ADI‐R values were missing for two participants. ADI‐R, Autism Diagnostic Interview‐Revised; ADOS, Autism Diagnostic Observation Schedule; ASD, autism spectrum disorder; CBCL, Child Behavior Checklist/4‐18R; CSS, calibrated severity score; IQ, intelligence quotient; NT, neurotypical controls; SRS‐16, Social Responsiveness Scale – 16 item version.

### Procedure

**Figure 1:**
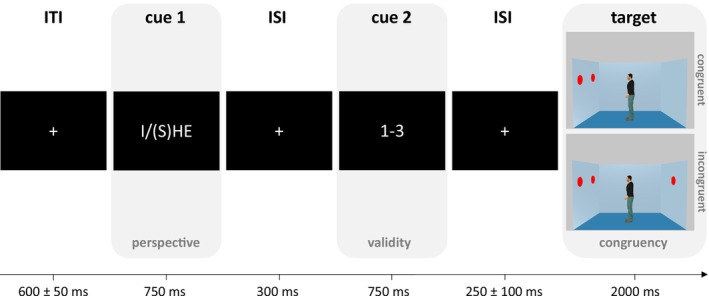
Illustration of a trial sequence. The *x*‐axis shows presentation durations for each stimulus. Cue 1 either consisted of the word ‘I’ or the word ‘HE/SHE’ (depending on the participant's sex). Cue 2 consisted of a number between 1 and 3. Trials were defined by the interaction of the perspective participants were required to attend (self/other; cue 1), the validity of the cued number of disks (valid/invalid; cue 2), and the congruency of the own and the avatar's perspective (congruent/incongruent; target). During target presentation, participants indicated via button press whether cue 2 was correct or incorrect. ISI, inter‐stimulus interval; ITI, inter‐trial interval

Participants completed an established VPT‐1 task (Figure 1; Samson et al., 2010) in an evenly lit testing room. After 10 practice trials, 240 trials were presented (3 blocks, ~5 min each). Each trial comprised two cues and a target picture. The first cue indicated the perspective the participant was required to attend (‘I’ or ‘HE’/‘SHE’; avatar's sex‐matched participants' reported sex). The second cue displayed a number (1–3), indicating a correct (valid) or incorrect (invalid) disk count visible from the cued perspective. The target depicted an avatar in a room with one to three disks. Participants indicated via button press (‘yes’/‘no’) whether the cued number matched the disks visible from the cued perspective. The number of disks visible to the participant could be congruent or incongruent with the number of disks visible to the avatar, yielding a factorial combination of perspective (self/other), cue validity (valid/invalid), and congruency (congruent/incongruent), with 30 trials per condition presented in randomized order.

### Apparatus

EEG was recorded with 64 sintered Ag/AgCl electrodes according to the extended International 10–20 system with FCz as recording reference and an additional electrode to record vertical EOG (~1 cm below right eye). Impedances were kept below 10 kΩ. Data were sampled at 1000 Hz and amplified using Brain Vision BrainAmp MR‐Plus amplifiers and Brain Vision Recorder software, with an online anti‐aliasing band pass filter (0.01–250 Hz, 12 dB/octave). Pupil responses were recorded with a Tobii Pro Spectrum eye‐tracker at 300 Hz.

### Preprocessing

Trials with invalid cues (50%) were excluded due to substantial neurophysiological activation differences to cue invalidity (Luck, Woodman, & Vogel, 2000; Wright, Geffen, & Geffen, 1995). Trials with incorrect responses were removed from all but response accuracy analyses. Trials with reaction time (RT) < 300 ms or > 2,000 ms (ASD: 1.40%, NT: 0.78%), and omitted responses (ASD: 2.17%, NT: 0.69%) were removed, yielding a median of 26.78 (ASD) and 28.03 (NT) trials per condition (*p* = .070). When multiple responses occurred, only the first was analyzed.

#### EEG

EEG data were preprocessed offline using BrainVision Analyzer 2.3, down‐sampled to 500 Hz, re‐referenced to the average, and high‐pass filtered at 0.1 Hz. Independent component analysis (ICA) based on 1 Hz high‐pass filtered data was used to manually identify and remove ocular and muscle artifacts, followed by a 40 Hz low‐pass filter. Data were epoched (−500 to 1700 ms; relative to target onset) and baseline corrected using the averaged 300 ms pre‐target interval. Trials were rejected using semi‐automated artifact detection (voltage step: 50 μV/ms, maximum difference of values: 200 μV, minimum activity: 0.5 μV). ERP electrodes and time windows were selected based on previous literature (Ferguson et al., 2018; McCleery et al., 2011; Peng et al., 2018; Sæther et al., 2021) and visual inspection of grand averages across groups. P200 (O1/O2/Oz, 175–275 ms), P3 (PO3/PO4/POz, 275–450 ms), and a negative‐going LFSW for each hemisphere due to left‐lateralized activation (left: AF7/F7/Fp1, right: AF8/F8/Fp2, 400–600 ms) were extracted. Mean amplitudes were computed at the trial level. Latencies were calculated at the subject level as 50% area latencies, referring to the timepoint when 50% of the integral within the respective time window was reached. Cases yielding multiple mathematical solutions were excluded (P200: *n* = 1, P3: *n* = 4, LFSW: *n* = 4).

#### Pupillary data

Invalid pupil sizes (<2 mm or >8 mm), dilation speed outliers (>median + 3 × median absolute deviation [MAD]), samples surrounding blinks (25 ms before and after 75–250 ms gaps), and outliers (>3 MAD deviation from smoothed trend line as identified by a two‐pass approach) were removed. Missing values of one eye were replaced by values of the other and corrected for the offset between both eyes. Data were smoothed with a 100 ms moving average, gaps ≤150 ms were interpolated, and pupil size was averaged across eyes. Pupillary responses during target presentation were baseline‐corrected by subtracting the mean pupil size during fixation cross presentation (150 ms) before target onset. SEPR was defined as mean trial‐level pupil response within three successive post‐target time windows (TW; TW‐1: 500–1,000 ms, TW‐2: 1,000–1,500 ms, TW‐3: 1,500–2,000 ms). Time points between 0 and 500 ms were mainly dominated by pupillary constriction resulting from cue‐to‐target luminance changes and thus excluded. To control for constriction‐related variance, SEPR was calculated as residuals from a linear regression model predicting the pupil response by constriction amplitude (minimum pupil response between 100 and 1,000 ms) and its latency.

### Statistical analysis

Statistical analyses were done using R version 4.4.2 (see: https://github.com/LeoniePolzer/VisualPerspectiveTakingAutism). Continuous variables were scaled and centered. Group differences were analyzed by mixed‐effect models including the main and interaction effects between group (ASD vs. NT), perspective (self vs. other) and congruency (congruent vs. incongruent) as fixed effects and subject as random intercept. Monte Carlo simulations based on the observed study design indicated adequate sensitivity for moderate three‐way interaction effects (standardized β ≥ 0.20, see Appendix S1). Random slopes were omitted due to convergence constraints. For LFSW analyses, hemisphere (left vs. right) was included in the interaction. For SEPR analyses, time window (TW‐1 vs. TW‐2 vs. TW‐3) was included in the interaction, and age as covariate due to group differences in the subsample. Linear mixed‐effect models were applied to examine group differences in RT, ERP amplitudes, and SEPR at the trial level, and to ERP latencies at the subject level. Response accuracy was analyzed on a trial level (response: correct vs. incorrect) using a binomial generalized linear mixed‐effects model. Post hoc testing contrasts on significant interaction effects were adjusted by false discovery rate (FDR) correction and are reported as standardized mean difference values (Δβ) or log‐odds difference (Δb), alongside unadjusted 95% confidence intervals. Interaction contrasts compared effect sizes of congruency effects that were observable in both groups.

To examine neurophysiological and behavioral associations, trial‐level data were aggregated at the subject level to harmonize data formats. Linear regression models analyzed combined effects of amplitudes and latencies on RT for each ERP and condition separately. LFSW models included interactions with hemisphere and applied Newey‐West correction to account for inter‐hemispheric correlations and residual autocorrelation. Similarly, autocorrelation across SEPR time windows was addressed by including a SEPR × time window interaction and applying Newey‐West correction. Shared contributions of pupillary and electrophysiological measures to RT were further investigated in the pupillometry subsample. SEPR during TW‐3 and its interaction with P3 signatures were included as predictors of RT for conditions in which both modalities independently predicted RT.

To examine associations between neurophysiological modalities, Pearson correlations were computed between ERP and pupillary measures. In addition, following the identification of reliable group differences in SEPR and LFSW amplitude, we conducted post hoc analyses to test whether SEPR as a global index of subcortically modulated cognitive effort contributed to condition‐sensitive LFSW effects. For this purpose, SEPR during TW‐3 and its interaction with group were entered as covariates in models of LFSW amplitude restricted to the pupillometry subsample.

## Results

We report highest order significant interactions, significant post hoc comparisons, and main effects not involved in interactions. Full models on group differences are provided in Tables S1–S18, alongside sensitivity analyses excluding participants with elevated ADHD symptoms and low‐trial ERP conditions.

### Behavioral task performance

**Figure 2:**
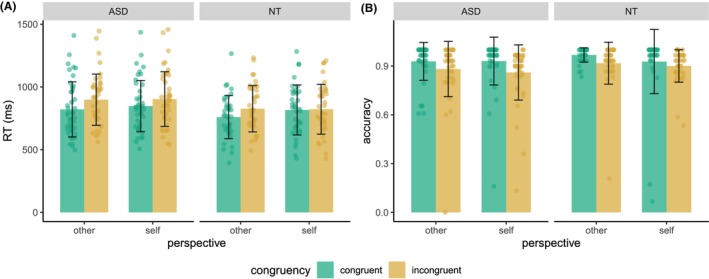
Distribution of the averaged‐per‐condition values of behavioral responses on a subject level. Bars and error bars represent means and standard deviations: (A) Shows averaged reaction times, (B) shows the number of correct responses standardized by the number of total trials. Within‐participant conditions with mean accuracy below 0.5 were observed in seven different participants. Importantly, no participant showed low accuracy in more than one condition, indicating intact task understanding alongside condition‐specific performance variability

Subject‐level performance distributions are shown in Figure 2.

#### Reaction time (RT)

RT was modulated by a perspective × congruency interaction (*F*(1, 8591.9) = 15.45, *p* < .001). This was qualified by slower RT for the other‐perspective than the self‐perspective in congruent trials (Δβ = 0.12, *p* < .001, 95% CI [0.07, 0.17]), and in incongruent compared to congruent trials across perspectives (self: Δβ = 0.10, *p* < .001, 95% CI [0.05, 0.15]; other: Δβ = 0.24, *p* < .001, 95% CI [0.19, 0.28]).

RT also varied by a group × congruency interaction (*F*(1, 8592.3) = 5.13, *p* = .023). Although both groups were slower for incongruent compared to congruent trials (ASD: Δβ = 0.21, *p* < .001, 95% CI [0.16, 0.25]; NT: Δβ = 0.13, *p* < .001, 95% CI [0.07, 0.18]), this effect was larger in autistic compared to neurotypical participants (Δβ = −0.08, *p* = .024, 95% CI [−0.15, −0.01]).

#### Response accuracy

Accuracy was modulated by a group × perspective × congruency interaction (χ^2^(1) = 8.98, *p* = .003). In post hoc contrasts, neurotypical compared to autistic participants showed a higher probability for correct responses when rating the other‐perspective during congruent (Δ*b* = 0.78, *p* = .035, 95% CI [0.14, 1.42]) but not incongruent trials (Δ*b* = 0.35, *p* = .281, 95% CI [−0.22, 0.92]). Moreover, neurotypical but not autistic participants showed a lower probability for correct responses when rating the self‐perspective compared to the other‐perspective in congruent trials (ASD: Δ*b* = 0.04, *p* = .812, 95% CI [−0.29, 0.37]; NT: Δ*b* = −0.95, *p* < .001, 95% CI [−1.36, −0.54]) but not in incongruent trials (ASD: Δ*b* = −0.27, *p* = .068, 95% CI [−0.53, −0.01]; NT: Δ*b* = −0.25, *p* = .173, 95% CI [−0.55, 0.06]). Both groups showed a higher probability for correct responses during congruent compared to incongruent trials when rating the other‐perspective (ASD: Δ*b* = −0.67, *p* < .001, 95% CI [−0.97, −0.37]; NT: Δ*b* = −1.10, *p* < .001, 95% CI [−1.51, −0.69]), and the self‐perspective (ASD: Δ*b* = −0.98, *p* < .001, 95% CI [−1.28, −0.68]; NT: Δ*b* = −0.40, *p* = .031, 95% CI [−0.71, −0.08]). However, this congruency effect was larger in autistic compared to neurotypical participants during ratings of the self‐perspective (Δ*b* = −0.58, *p* = .008, 95% CI [−1.02, −0.15]) but not the other‐perspective (Δ*b* = 0.43, *p* = .093, 95% CI [−0.07, 0.94]).

### Event‐related potentials (ERPs)

#### P200

**Figure 3:**
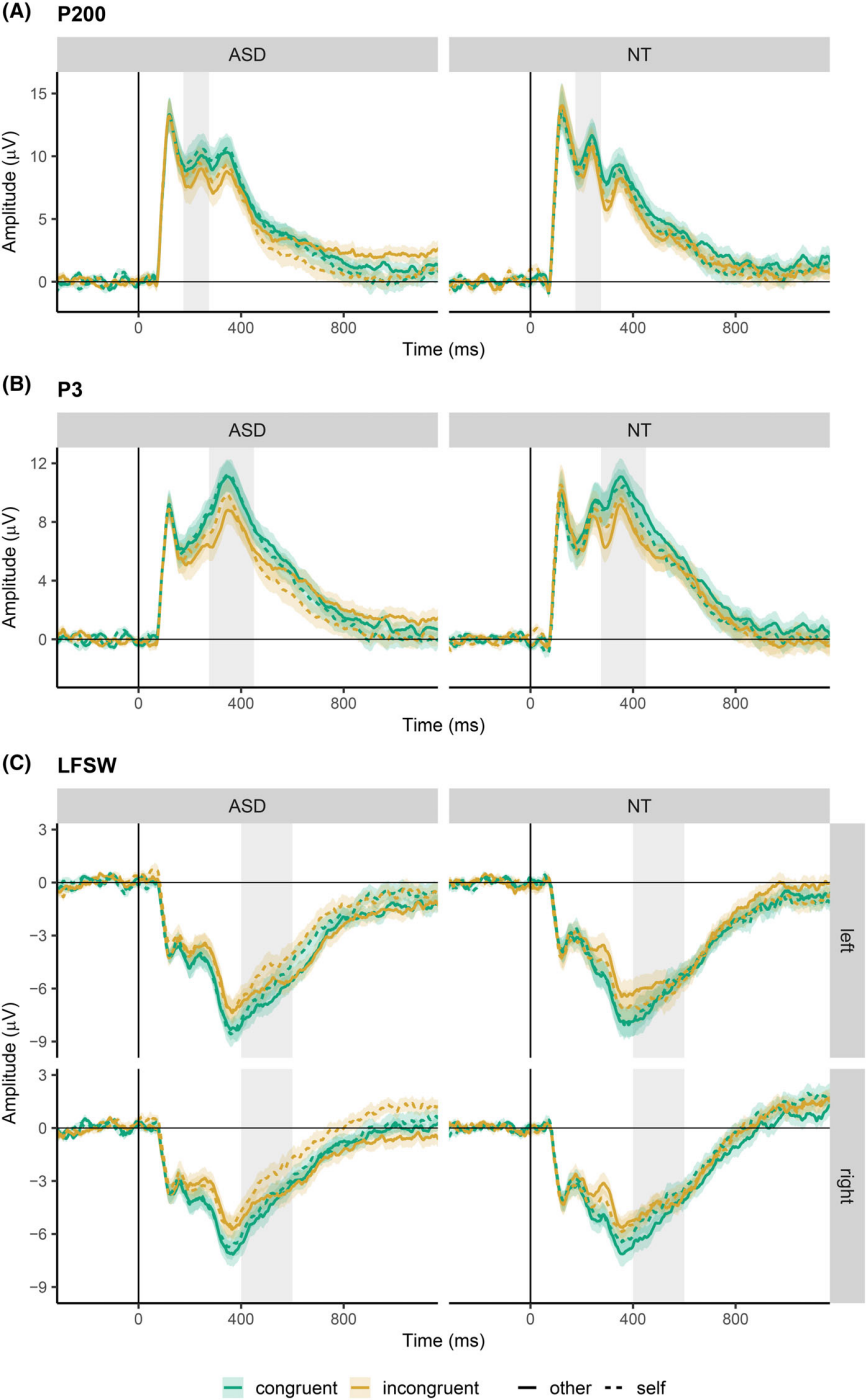
Event‐related potential waveforms per group and condition during target presentation: (A) shows aggregated waveforms over occipito‐parietal electrodes (P200), (B) shows aggregated waveforms over centro‐parietal electrodes (P3), and (C) shows aggregated waveforms over frontal electrodes, separated by hemisphere (LFSW). Shaded areas indicate the standard error of the mean (*SEM*) across participants at each time point. Gray background boxes mark the predefined time windows used for component quantification. In line with previous studies, LFSW characteristics comprised slowly decreasing activation after an earlier negative peak and left‐lateralization (Ferguson et al., 2018; Sæther et al., 2021)

Congruency influenced P200 *amplitude*, with larger amplitudes for congruent compared to incongruent trials (*F*(1, 8103.2) = 16.39, *p* < .001, Δβ = 0.07, 95% CI [0.04, 0.10]; see Figure 3A), with no group effects on amplitude or latency.

#### P3

Congruent trials elicited larger P3 *amplitudes* than incongruent trials (*F*(1, 8034.4) = 87.23, *p* < .001, Δβ = 0.17, 95% CI [0.14, 0.21]; see Figure 3B). P3 *latency* was longer for the other‐perspective compared to the self‐perspective (*F*(1, 234.31) = 6.46, *p* = .012, Δβ = 0.23, 95% CI [0.05, 0.41]). No group effects were observed.

#### LFSW

LFSW *amplitude* was more negative (i.e., larger) in the left than the right hemisphere (*F*(1, 16,143.3) = 227.75, *p* < .001, Δβ = −0.22, 95% CI [−0.24, −0.19]). Crucially, LFSW amplitude varied by a group × perspective × congruency interaction (*F*(1, 16,147.3) = 4.16, *p* = .041; see Figure 3C). Autistic but not neurotypical participants showed a less negative LFSW amplitude for the self‐perspective relative to the other‐perspective in incongruent (ASD: Δβ = 0.11, *p* < .001, 95% CI [0.04, 0.16]; NT: Δβ = −0.033, *p* = .975, 95% CI [−0.09, 0.02]) and congruent trials (ASD: Δβ = 0.08, *p* = .015, 95% CI [0.02, 0.13]; NT: Δβ = 0.05, *p* = .114, 95% CI [−0.001, 0.11]), with no difference between congruency conditions in autism (Δβ = −0.03, *p* = .464, 95% CI [−0.11, 0.05]). Moreover, autistic but not neurotypical participants showed a less negative LFSW amplitude when rating incongruent compared to congruent trials in the self‐perspective (ASD: Δβ = 0.12, *p* < .001, 95% CI [0.06, 0.17]; NT: Δβ = 0.01, *p* = .821, 95% CI [−0.05, 0.07]), whereas both groups showed this pattern when rating the other‐perspective (ASD: Δβ = 0.09, *p* = .006, 95% CI [0.03, 0.14]; NT: Δβ = 0.10, *p* = .003, 95% CI [0.04, 0.15]).

The LFSW *latency* was influenced by a group × perspective × congruency × hemisphere interaction (*F*(1,549.22) = 6.39, *p* = .012). After correction, only one contrast remained significant, with autistic participants showing longer right‐hemispheric LFSW latencies for incongruent compared to congruent trials during other‐perspective ratings (Δβ = 0.69, *p* = .006, 95% CI [0.32, 1.05]).

### Stimulus‐evoked pupil responses (SEPR)

In the pupillometry subsample, SEPR varied by a congruency × time window interaction (*F*(2,15143.6) = 4.04, *p* = .018), with larger SEPR for incongruent than congruent trials during TW‐2 and TW‐3 (both: Δβ = 0.07, *p* = .003, 95% CI [0.03, 0.12]) and increases from TW‐1 to TW‐2 in congruent (Δβ = 0.89, *p* < .001, 95% CI [0.84, 0.93]) and incongruent trials (Δβ = 0.97, *p* < .001, 95% CI [0.92, 1.02]).

**Figure 4:**
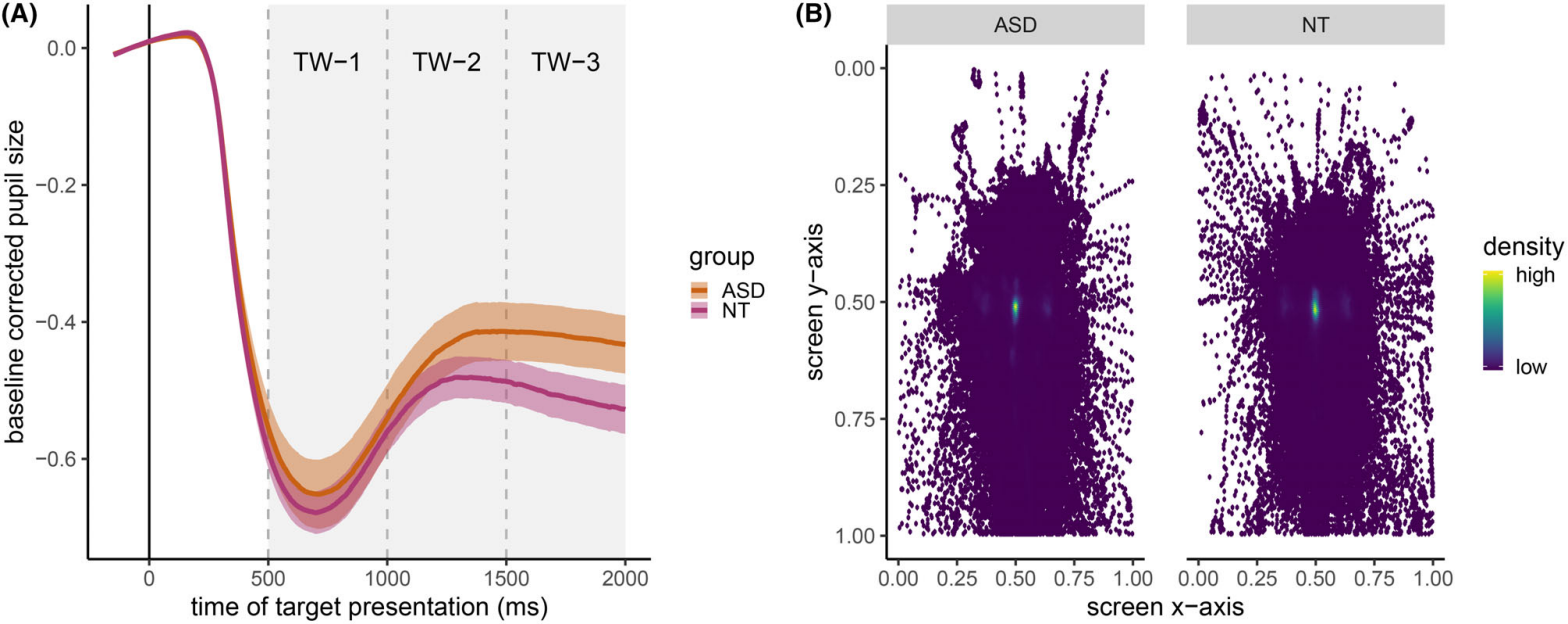
Pupil responses and gaze behavior during target presentation in the pupillometry subsample: (A) shows the baseline‐corrected progression of the pupil size during target presentation. Values within the three different time windows (TW‐1, TW‐2, TW‐3) were subsequently corrected for the amplitude and latency of the pupillary constriction. (B) shows the density of gaze observations on the screen. Gaze behavior was similarly distributed across the screen, with a clear focus to the center of the screen, where the avatar was presented

Crucially, there was a significant group × time window interaction (*F*(2,15144.7) = 36.72, *p* < .001). Post hoc contrasts revealed that autistic relative to neurotypical participants exhibited a larger SEPR in TW‐3 (Δβ = 0.26, *p* = .029, 95% CI [−0.48, −0.05]). Moreover, while SEPR increased from TW‐1 to TW‐2 in both groups (ASD: Δβ = 0.98, *p* < .001, 95% CI [0.93, 1.02]; NT: Δβ = 0.88, *p* < .001, 95% CI [0.83, 0.92]), autistic participants showed a further increase from TW‐2 to TW‐3 (Δβ = 0.13, *p* < .001, 95% CI [0.08, 0.18]), whereas NT participants showed a decline (Δβ = −0.05, *p* = .044, 95% CI [−0.10, −0.004]). A trend toward a group × congruency × time window interaction (*F*(2, 15,143.6) = 2.99, *p* = .050) pointed toward group differences being marginally more pronounced in incongruent trials. Correcting for in‐trial pupil constriction amplitude and latency as covariates instead of residualizing effects out (see Table S19) and for potential gaze influences by adding gaze deviation from the center of the screen as a covariate yielded comparable results (see Table S20 and Figure 4B).

### Behavioral relevance of neurophysiological markers

P200 signatures did not predict RT in any condition. Larger P3 amplitudes predicted slower RT in the other‐perspective in congruent trials (β = .027, *p* = .014, 95% CI [0.05, 0.49]) and in incongruent trials (β = 0.27, *p* = .017, 95% CI [0.05, 0.49]), while both larger P3 amplitudes (β = 0.22, *p* = .037, 95% CI [0.01, 0.43]) and longer P3 latencies (β = 0.38, *p* < .001, 95% CI [0.17, 0.59]) were associated with slower RT in the self‐perspective in incongruent trials. Longer right‐hemispheric LFSW latencies predicted slower RT in the other‐perspective in incongruent trials (β = 0.21, *p* = .037, 95% CI [0.01, 0.41]).

In the pupillometry subsample, smaller SEPR during TW‐3 predicted slower RT during self‐perspective ratings in congruent (β = 0.34, *p* = .015, 95% CI [0.07, 0.61]) and incongruent trials (β = 0.32, *p* = .014, 95% CI [0.07, 0.58]).

As both P3 signatures and SEPR during TW‐3 predicted RT in the self‐perspective in incongruent trials, we examined distinct and shared influences on RT in this condition. Replicating full sample effects, larger P3 amplitude and longer P3 latency predicted slower RT in the subsample when controlling for their mutual influences (amplitude: β = 0.37, *p* = .004, 95% CI [0.12, 0.62], latency: β = 0.48, *p* < .001, 95% CI [0.23, 0.72]). Adding SEPR during TW‐3 and its interaction with P3 signatures revealed an additional main effect of SEPR (β = 0.28, *p* = .023, 95% CI [0.04, 0.52]), alongside P3 latency (β = 0.36, *p* = .005, 95% CI [0.11, 0.61]), whereas P3 amplitude no longer explained unique variance (β = 0.22, *p* = .106, 95% CI [−0.05, 0.50]). No interaction effects were observed.

### Multimodal associations between neurophysiological markers

In the pupillometry subsample, ERP measures showed small to moderate associations with SEPR, most pronounced between SEPR during later windows (TW‐2 and TW‐3) and P200 and P3 amplitudes (Table 2). After FDR correction, significant correlations were confined to the incongruent self‐perspective condition.

**Table 2:** Pearson correlations between SEPR and ERPs within task conditions in the pupillometry subsample

	P200 amp	P200 lat	P3 amp	P3 lat	LFSW amp left	LFSW lat left	LFSW amp right	LFSW lat right
Congruent – self								
SEPR‐TW1	−.21	−.00	−.16	−.16	.05	.07	.11	−.09
SEPR‐TW2	.27	−.11	.24	−.11	.00	.16	.01	.20
SEPR‐TW3	.30*	−.11	.20	.03	.07	.22	.05	.21
Congruent – other								
SEPR‐TW1	−.28*	−.04	−.16	−.13	.14	−.03	.24	−.14
SEPR‐TW2	.31*	.03	.32*	.11	−.19	.21	−.21	.17
SEPR‐TW3	.35**	−.01	.24	.13	−.04	.14	−.20	.25
Incongruent – self								
SEPR‐TW1	−.16	−.11	−.08	.06	.18	−.15	.13	−.15
SEPR‐TW2	.32*	.15	.31*	−.02	−.10	.13	−.28*	−.06
SEPR‐TW3	.40**	.08	.30*	.04	.03	.16	−.17	−.05
Incongruent – other								
SEPR‐TW1	−.16	−.12	−.16	.03	.06	−.09	.06	−.03
SEPR‐TW2	.39**	.02	.43**	.21	−.19	.11	−.26	.15
SEPR‐TW3	.48***	−.11	.36**	.30*	−.09	.07	−.21	.17

Asterisks indicate uncorrected significance levels (*p < .05, **p < .01, ***p < .001). Values shown in bold indicate correlations that remain significant after false discovery rate (FDR) correction within condition. Amp, amplitude; lat, latency; LFSW, late‐frontal slow wave; SEPR, stimulus‐evoked pupillary response; TW, time window.

To further examine associations between neurophysiological markers that differed reliably between groups, we tested effects of SEPR on the condition‐sensitive patterns in LFSW amplitude. Including SEPR during TW‐3 in the LFSW amplitude model did not change the general pattern of results, and neither SEPR nor its interaction with group predicted the LFSW amplitude (see Table S21).

## Discussion

This study investigated VPT and concurrent effects of perspective incongruency in autistic youth. Extending prior behavioral work, we combined ERPs and pupillometry to characterize underlying mechanisms of behaviorally observable phenomena. Replicating findings from neurotypical adults, adolescents showed detrimental effects of perspective incongruency on behavior – particularly when rating the avatar's perspective – alongside convergent ERP modulations, demonstrating that the paradigm and neurophysiological measures capture similar processes in youth. In autism, we report altered VPT, pronounced perspective incongruency costs and neurophysiological processing differences.

### Behavioral performance

Unlike prior VPT studies reporting no accuracy differences between autistic and neurotypical adults (Doi et al., 2020; Schwarzkopf et al., 2014; Tei et al., 2019), we found a reduced accuracy in autistic youth during avatar‐ but not self‐perspective judgments. Consistent with reports of altered VPT‐2 in autistic adolescents but not adults (Conson et al., 2015; O'Connor et al., 2023), diverging findings may indicate age‐dependent difficulties in VPT‐1 in autism, although methodological differences should be acknowledged.

Notably, neurotypical participants showed higher accuracy when reporting the avatar's perspective compared to their own, potentially reflecting the benefit of using a salient directional cue versus the cost of suppressing it during self‐perspective judgments. In line with altered perspective calculations depending on their relevance to task goals (Todd, Cameron, & Simpson, 2021), increased salience of social demands in the context of an autism‐focused study may additionally have shifted the cognitive set toward taking over others' perspectives. Importantly, autistic participants did not show this pattern, indicating a reduced suppression of directional cues or a reduced cognitive emphasis on adopting others' perspectives in a social task. While RT effects in favor of the avatar's perspective have been reported before (Samson et al., 2010), these patterns in behavioral accuracy require replication.

Contrasting previous findings in adults (Doi et al., 2020; Schwarzkopf et al., 2014; Tei et al., 2019), we additionally found a larger incongruency effect on RT in autistic compared to neurotypical participants. This was accompanied by a larger incongruency‐related decrease in response accuracy during ratings of their own perspective in autistic compared to neurotypical participants, supporting elevated difficulties to disregard irrelevant stimuli in autistic youth.

### Neurophysiological activation

As the main focus of the study, we examined neurophysiological signatures during VPT. We found an autism‐specific reduction in LFSW amplitude for the own relative to the avatar's perspective. Late‐frontal ERPs have been linked to top‐down perspective management and post‐decisional response selection (McCleery et al., 2011; Sæther et al., 2021; Strandburg et al., 1996). Accordingly, findings indicate an increased top‐down recruitment during VPT in autism, potentially reflecting a less automatized VPT process. Extending recent findings of frontal differences limited to VPT‐2 in autistic adolescents (Seymour et al., 2025), our results show that frontal processing differences are also observable during lower level VPT‐1. Moreover, while LFSW amplitudes were modulated by incongruency during avatar‐perspective ratings in both groups, only autistic participants showed similar effects during self‐perspective ratings. We assume that incongruency may distract neurotypical youth only under a second task requirement such as perspective taking, whereas a lower threshold for distraction in autism may lead to a decreased certainty during top‐down signaling.

In a subsample, autistic participants showed larger and a more sustained SEPR than neurotypical participants, with a nonsignificant trend toward additional effects of perspective incongruency, likely due to limited power. These findings indicate an increased cognitive effort in autism. Due to marginal incongruency effects, we assume this to be driven by pronounced difficulties during perspective ratings in contrast to general task demands such as motor response preparation.

We further examined associations between neurophysiological markers and behavioral performance. Shorter P3 and LFSW latencies were associated with faster responses, particularly under perspective incongruency, when demands on selective attention are highest. P3 amplitude showed reduced explanatory power for RT when SEPR was accounted for, indicating that P3 amplitude and SEPR capture partially overlapping aspects of attentional effort, whereas P3 latency may rather reflect perspective selection. SEPR predicted RT selectively during self‐perspective judgments, aligning with proposals that SEPR is largely driven by decision formation processes (de Gee, Knapen, & Donner, 2014), while perspective selection processes may override this association during VPT. Importantly, autism‐related differences in SEPR did not account for group differences in LFSW amplitude. Altered subcortical indices of cognitive effort and frontal slow‐wave dynamics are hence unlikely to reflect a single shared mechanism and are supported as independent contributions to perspective ratings in autism.

### Integrated findings of perspective incongruency

As hypothesized, autistic participants showed larger effects of perspective incongruency across behavioral and neurophysiological measures. While incongruency during VPT has previously been regarded as provoking involuntary perspective calculations (Samson et al., 2010), recent accounts challenge this view by emphasizing domain‐general mechanisms (Heyes, 2014). This is supported by incongruency effects for nonsocial directional cues (Gardner & Thorn, 2025; Santiesteban, Catmur, Hopkins, Bird, & Heyes, 2014), which may however be amplified by social content (Kragh Nielsen, Slade, Levy, & Holmes, 2015). We propose that increased incongruency effects in autism predominantly reflect difficulties to selectively inhibit the irrelevant information of the avatar (self‐perspective) or additional disks (other‐perspective). While social contributions cannot be excluded, group differences emerging specifically in activity linked to top‐down response selection (LFSW) rather than self‐other distinctions (P3 latency) suggest a stronger involvement of domain‐general mechanisms. This interpretation aligns with evidence of increased VPT alterations in autism under higher cognitive demands (Pearson et al., 2013; Reed, 2002; Seymour et al., 2025), and provides a parsimonious explanation for similar autism‐related interference effects in nonsocial contexts (Keehn et al., 2016; Murphy, Foxe, Peters, & Molholm, 2014). We assume that an increased demand for selective attention affects the general ability to process visual‐perceptual input in autism, with downstream effects on social cognition.

### Limitations

A general limitation is the difficulty to interpret specific contributions to VPT performance, as effects are confounded with processing of directional cues. Comparing social to nonsocial cues cannot completely isolate social influences due to diverging nonsocial cue characteristics, such as the difficulty to extract indicated directions. Future neurophysiological or functional neuroimaging studies may better dissociate social and domain‐general contributions.

Moreover, other characteristics of the paradigm like switching perspectives in‐between trials may have influenced results. Yet, lacking effects within autistic participants (Doi et al., 2020) and on group comparisons (Schwarzkopf et al., 2014) make this explanation unlikely.

The autistic sample was not representative of the full spectrum with respect to intellectual functioning, as groups were IQ‐matched to isolate autism‐specific effects in a cognitively demanding task. Additionally, although excluding autistic participants with clinically significant ADHD symptoms did not qualitatively alter main results, future studies may include additional groups to directly address effects of co‐occurring ADHD.

## Conclusion

In summary, our findings demonstrate atypical VPT processing in autistic youth. Differential neurophysiological patterns indicate an altered top‐down recruitment during VPT and an increased cognitive effort during perspective ratings. Additionally, difficulties with selective attention requirements in autism are highlighted as a general mechanism during the recount of visual input, which concurrently affects visually primed social cognition demands. Findings contextualize social cognition differences within a broader framework of domain‐general cognitive contributions in neurodiversity.

## Ethical considerations

Primary caregivers and participants gave their written informed consent. The project was approved by the ethics committee of the Medical Faculty at the University Hospital Frankfurt (votum 416/17; 16th October 2017).

**Key points**

**What's known?**

- Altered social cognition in autism is influenced by domain‐general functions, such as selective attention.

**What's new?**

- This study delineated underlying mechanisms of visual perspective taking (VPT) by assessing neurophysiological activity and manipulating selective attention demands in autistic compared to neurotypical youth.
- Autistic youth showed diminished VPT, accompanied by atypical frontal top‐down modulation, increased cognitive effort as indicated by pupil responses, and pronounced difficulties in deploying selective attention.

**What's relevant?**

- Neurophysiological markers are emphasized as promising tools to specify mechanisms of social cognition differences in neurodiversity.

## Supporting information


**Table S1.** Reaction times as predicted by group and task conditions.
**Table S2.** Response accuracy as predicted by group and task conditions (binomial generalized linear mixed‐effects model).
**Table S3.** Sensitivity analysis: Reaction times after exclusion of *n* = 7 participants fulfilling ICD‐10 ADHD criteria (FBB‐ADHD).
**Table S4.** Sensitivity analysis: Response accuracy after exclusion of *n* = 7 participants fulfilling ICD‐10 ADHD criteria (FBB‐ADHD).
**Table S5.** P200 mean amplitude as predicted by group and task conditions.
**Table S6.** P200 50% area latency as predicted by group and task conditions.
**Table S7.** P3 mean amplitude as predicted by group and task conditions.
**Table S8.** P3 50% area latency as predicted by group and task conditions.
**Table S9.** LFSW mean amplitude as predicted by group and task conditions.
**Table S10.** LFSW 50% area latency as predicted by group and task conditions.
**Table S11.** Sensitivity analysis: P200 mean amplitude after exclusion of within‐participant conditions with < 15 trials.
**Table S12.** Sensitivity analysis: P200 50% area latency after exclusion of within‐participant conditions with < 15 trials.
**Table S13.** Sensitivity analysis: P3 mean amplitude after exclusion of within‐participant conditions with < 15 trials.
**Table S14.** Sensitivity analysis: P3 50% area latency after exclusion of within‐participant conditions with < 15 trials.
**Table S15.** Sensitivity analysis: LFSW mean amplitude after exclusion of within‐participant conditions with < 15 trials.
**Table S16.** Sensitivity analysis: LFSW 50% area latency after exclusion of within‐participant conditions with < 15 trials.
**Table S17.** Sensitivity analysis: LFSW amplitude after exclusion of *n* = 7 participants fulfilling ICD‐10 ADHD criteria (FBB‐ADHD).
**Table S18.** SEPR as predicted by group and task conditions (*n* = 55).
**Table S19.** Nonresidualized pupil response as predicted by group, task conditions and pupil constriction covariates.
**Table S20.** SEPR as predicted by group, task conditions and additional covariate of gaze deviation from the center of the screen.
**Table S21.** Group differences in LFSW amplitude with additional predictor of SEPR (TW‐3) in the pupillometry subsample.
**Appendix S1.** Simulation‐based power analyses.

## Data Availability

The data that support the findings of this study are available from the corresponding author upon reasonable request.
