# Supplementary material for: Attenuated selective attention during visual perspective taking in autism: A combined behavior, ERP, and pupillometry study
Source: J Child Psychol Psychiatry. 2026 Apr 16;67(9):1500–12. doi: 10.1111/jcpp.70156 (PMC13450282; doi:10.1111/jcpp.70156)
Supplement: Supplementary file 1 [file JCPP-67-1500-s001.docx]

**Attenuated Selective Attention during Visual Perspective Taking in Autism: A Combined Behavior, ERP, and Pupillometry Study**

**Supporting Information**

Abbreviations: late frontal slow wave (LFSW), stimulus-evoked pupillary response (SEPR)

**Content**

[**Behavioral task performance**	3](#_Toc226974921)

[Table S1. Reaction times as predicted by group and task conditions.	3](#_Toc226974922)

[Table S2. Response accuracy as predicted by group and task conditions (binomial generalized linear mixed-effects model).	3](#_Toc226974923)

[Table S3. Sensitivity analysis: Reaction times after exclusion of n=7 participants fulfilling ICD-10 ADHD criteria (FBB-ADHD).	3](#_Toc226974924)

[Table S4. Sensitivity analysis: Response accuracy after exclusion of n=7 participants fulfilling ICD-10 ADHD criteria (FBB-ADHD).	3](#_Toc226974925)

[**Event-related potentials**	4](#_Toc226974926)

[Table S5. P200 mean amplitude as predicted by group and task conditions.	4](#_Toc226974927)

[Table S6. P200 50% area latency as predicted by group and task conditions.	4](#_Toc226974928)

[Table S7. P3 mean amplitude as predicted by group and task conditions.	4](#_Toc226974929)

[Table S8. P3 50% area latency as predicted by group and task conditions.	4](#_Toc226974930)

[Table S9. LFSW mean amplitude as predicted by group and task conditions.	5](#_Toc226974931)

[Table S10. LFSW 50% area latency as predicted by group and task conditions.	5](#_Toc226974932)

[Table S11. Sensitivity analysis: P200 mean amplitude after exclusion of within-participant conditions with < 15 trials	6](#_Toc226974933)

[Table S12. Sensitivity analysis: P200 50% area latency after exclusion of within-participant conditions with < 15 trials	6](#_Toc226974934)

[Table S13. Sensitivity analysis: P3 mean amplitude after exclusion of within-participant conditions with < 15 trials	6](#_Toc226974935)

[Table S14. Sensitivity analysis: P3 50% area latency after exclusion of within-participant conditions with < 15 trials	6](#_Toc226974936)

[Table S15. Sensitivity analysis: LFSW mean amplitude after exclusion of within-participant conditions with < 15 trials	7](#_Toc226974937)

[Table S16. Sensitivity analysis: LFSW 50% area latency after exclusion of within-participant conditions with < 15 trials	7](#_Toc226974938)

[Table S17. Sensitivity analysis: LFSW amplitude after exclusion of n=7 participants fulfilling ICD-10 ADHD criteria (FBB-ADHD)	8](#_Toc226974939)

[**Pupillary responses**	9](#_Toc226974940)

[Table S18. SEPR as predicted by group and task conditions (n=55).	9](#_Toc226974941)

[Table S19. Non-residualized pupil response as predicted by group, task conditions and pupil constriction covariates.	9](#_Toc226974942)

[Table S20. SEPR as predicted by group, task conditions and additional covariate of gaze deviation from the center of the screen.	10](#_Toc226974943)

[**Associative analyses**	11](#_Toc226974944)

[Table S21. Group differences in LFSW amplitude with additional predictor of SEPR (TW-3) in the pupillometry subsample.	11](#_Toc226974945)

[**Additional information**	12](#_Toc226974946)

[Appendix S1. Simulation-based power analyses	12](#_Toc226974947)

# **Behavioral task performance**

## Table S1. Reaction times as predicted by group and task conditions.

|  | Sum Sq | Mean Sq | NumDF | DenDF | F value | p |
| --- | --- | --- | --- | --- | --- | --- |
| group | 1.534 | 1.534 | 1 | 78.691 | 2.358 | 0.129 |
| perspective | 6.162 | 6.162 | 1 | 8593.819 | 9.474 | 0.002 |
| congruency | 60.941 | 60.941 | 1 | 8592.304 | 93.700 | 0.000 |
| group:perspective | 0.972 | 0.972 | 1 | 8593.819 | 1.495 | 0.221 |
| group:congruency | 3.339 | 3.339 | 1 | 8592.304 | 5.134 | 0.023 |
| perspective:congruency | 10.047 | 10.047 | 1 | 8591.898 | 15.448 | 0.000 |
| group:perspective:congruency | 0.181 | 0.181 | 1 | 8591.898 | 0.279 | 0.598 |

## Table S2. Response accuracy as predicted by group and task conditions (binomial generalized linear mixed-effects model).

|  | Chisq | Df | p |
| --- | --- | --- | --- |
| group | 1.274 | 1 | 0.259 |
| perspective | 13.419 | 1 | 0.000 |
| congruency | 83.659 | 1 | 0.000 |
| group:perspective | 4.377 | 1 | 0.036 |
| group:congruency | 0.830 | 1 | 0.362 |
| perspective:congruency | 0.352 | 1 | 0.553 |
| group:perspective:congruency | 8.979 | 1 | 0.003 |

## Table S3. Sensitivity analysis: Reaction times after exclusion of n=7 participants fulfilling ICD-10 ADHD criteria (FBB-ADHD).

|  | Sum Sq | Mean Sq | NumDF | DenDF | F value | p |
| --- | --- | --- | --- | --- | --- | --- |
| group | 0.221 | 0.221 | 1 | 71.745 | 0.329 | 0.568 |
| perspective | 6.325 | 6.325 | 1 | 8021.190 | 9.417 | 0.002 |
| congruency | 65.365 | 65.365 | 1 | 8020.241 | 97.323 | 0.000 |
| group:perspective | 0.950 | 0.950 | 1 | 8021.190 | 1.414 | 0.234 |
| group:congruency | 4.294 | 4.294 | 1 | 8020.241 | 6.394 | 0.011 |
| perspective:congruency | 11.533 | 11.533 | 1 | 8019.520 | 17.172 | 0.000 |
| group:perspective:congruency | 0.048 | 0.048 | 1 | 8019.520 | 0.071 | 0.789 |

## Table S4. Sensitivity analysis: Response accuracy after exclusion of n=7 participants fulfilling ICD-10 ADHD criteria (FBB-ADHD).

|  | Chisq | Df | p |
| --- | --- | --- | --- |
| group | 0.036 | 1 | 0.849 |
| perspective | 26.286 | 1 | 0.000 |
| congruency | 85.840 | 1 | 0.000 |
| group:perspective | 0.143 | 1 | 0.706 |
| group:congruency | 5.086 | 1 | 0.024 |
| perspective:congruency | 0.759 | 1 | 0.384 |
| group:perspective:congruency | 8.765 | 1 | 0.003 |

# **Event-related potentials**

## Table S5. P200 mean amplitude as predicted by group and task conditions.

|  | Sum Sq | Mean Sq | NumDF | DenDF | F value | p |
| --- | --- | --- | --- | --- | --- | --- |
| group | 0.055 | 0.055 | 1 | 78.975 | 0.096 | 0.757 |
| perspective | 1.139 | 1.139 | 1 | 8103.619 | 1.999 | 0.157 |
| congruency | 9.338 | 9.338 | 1 | 8103.177 | 16.392 | 0.000 |
| group:perspective | 1.401 | 1.401 | 1 | 8103.619 | 2.459 | 0.117 |
| group:congruency | 0.658 | 0.658 | 1 | 8103.177 | 1.155 | 0.283 |
| perspective:congruency | 1.569 | 1.569 | 1 | 8102.686 | 2.755 | 0.097 |
| group:perspective:congruency | 0.481 | 0.481 | 1 | 8102.686 | 0.845 | 0.358 |

## Table S6. P200 50% area latency as predicted by group and task conditions.

|  | Sum Sq | Mean Sq | NumDF | DenDF | F value | p |
| --- | --- | --- | --- | --- | --- | --- |
| group | 1.669 | 1.669 | 1 | 79.266 | 2.829 | 0.097 |
| perspective | 0.008 | 0.008 | 1 | 235.589 | 0.014 | 0.906 |
| congruency | 0.038 | 0.038 | 1 | 235.589 | 0.065 | 0.799 |
| group:perspective | 1.683 | 1.683 | 1 | 235.589 | 2.853 | 0.093 |
| group:congruency | 0.929 | 0.929 | 1 | 235.589 | 1.574 | 0.211 |
| perspective:congruency | 0.045 | 0.045 | 1 | 235.589 | 0.076 | 0.783 |
| group:perspective:congruency | 0.076 | 0.076 | 1 | 235.589 | 0.128 | 0.721 |

## Table S7. P3 mean amplitude as predicted by group and task conditions.

|  | Sum Sq | Mean Sq | NumDF | DenDF | F value | p |
| --- | --- | --- | --- | --- | --- | --- |
| group | 0.000 | 0.000 | 1 | 79.050 | 0.000 | 0.994 |
| perspective | 0.777 | 0.777 | 1 | 8035.878 | 1.144 | 0.285 |
| congruency | 59.289 | 59.289 | 1 | 8034.396 | 87.226 | 0.000 |
| group:perspective | 0.375 | 0.375 | 1 | 8035.878 | 0.552 | 0.458 |
| group:congruency | 0.007 | 0.007 | 1 | 8034.396 | 0.011 | 0.918 |
| perspective:congruency | 1.820 | 1.820 | 1 | 8033.692 | 2.677 | 0.102 |
| group:perspective:congruency | 0.094 | 0.094 | 1 | 8033.692 | 0.138 | 0.710 |

## Table S8. P3 50% area latency as predicted by group and task conditions.

|  | Sum Sq | Mean Sq | NumDF | DenDF | F value | p |
| --- | --- | --- | --- | --- | --- | --- |
| group | 0.664 | 0.664 | 1 | 79.478 | 0.987 | 0.323 |
| perspective | 4.348 | 4.348 | 1 | 234.306 | 6.462 | 0.012 |
| congruency | 0.919 | 0.919 | 1 | 234.306 | 1.365 | 0.244 |
| group:perspective | 0.255 | 0.255 | 1 | 234.306 | 0.379 | 0.539 |
| group:congruency | 1.035 | 1.035 | 1 | 234.306 | 1.539 | 0.216 |
| perspective:congruency | 0.008 | 0.008 | 1 | 234.306 | 0.011 | 0.916 |
| group:perspective:congruency | 2.333 | 2.333 | 1 | 234.306 | 3.467 | 0.064 |

## Table S9. LFSW mean amplitude as predicted by group and task conditions.

|  | Sum Sq | Mean Sq | NumDF | DenDF | F value | p |
| --- | --- | --- | --- | --- | --- | --- |
| group | 0.424 | 0.424 | 1 | 79.231 | 0.512 | 0.477 |
| perspective | 10.431 | 10.431 | 1 | 16152.626 | 12.582 | 0.000 |
| congruency | 24.061 | 24.061 | 1 | 16149.035 | 29.022 | 0.000 |
| hemisphere | 188.815 | 188.815 | 1 | 16143.257 | 227.745 | 0.000 |
| group:perspective | 6.386 | 6.386 | 1 | 16152.626 | 7.703 | 0.006 |
| group:congruency | 2.278 | 2.278 | 1 | 16149.035 | 2.748 | 0.097 |
| perspective:congruency | 0.865 | 0.865 | 1 | 16147.300 | 1.044 | 0.307 |
| group:hemisphere | 2.897 | 2.897 | 1 | 16143.257 | 3.494 | 0.062 |
| perspective:hemisphere | 1.410 | 1.410 | 1 | 16143.257 | 1.701 | 0.192 |
| congruency:hemisphere | 0.111 | 0.111 | 1 | 16143.257 | 0.134 | 0.715 |
| group:perspective:  congruency | 3.449 | 3.449 | 1 | 16147.300 | 4.161 | 0.041 |
| group:perspective:  hemisphere | 1.419 | 1.419 | 1 | 16143.257 | 1.711 | 0.191 |
| group:congruency:  hemisphere | 0.094 | 0.094 | 1 | 16143.257 | 0.114 | 0.736 |
| perspective:congruency:  hemisphere | 0.786 | 0.786 | 1 | 16143.257 | 0.947 | 0.330 |
| group:perspective:  congruency:hemisphere | 0.092 | 0.092 | 1 | 16143.257 | 0.111 | 0.738 |

## Table S10. LFSW 50% area latency as predicted by group and task conditions.

|  | Sum Sq | Mean Sq | NumDF | DenDF | F value | p |
| --- | --- | --- | --- | --- | --- | --- |
| group | 0.006 | 0.006 | 1 | 79.047 | 0.008 | 0.928 |
| perspective | 2.071 | 2.071 | 1 | 549.518 | 2.834 | 0.093 |
| congruency | 12.861 | 12.861 | 1 | 549.543 | 17.595 | 0.000 |
| hemisphere | 2.070 | 2.070 | 1 | 549.229 | 2.832 | 0.093 |
| group:perspective | 0.103 | 0.103 | 1 | 549.518 | 0.141 | 0.708 |
| group:congruency | 0.038 | 0.038 | 1 | 549.543 | 0.052 | 0.820 |
| perspective:congruency | 0.676 | 0.676 | 1 | 549.543 | 0.925 | 0.337 |
| group:hemisphere | 0.040 | 0.040 | 1 | 549.229 | 0.054 | 0.816 |
| perspective:hemisphere | 1.898 | 1.898 | 1 | 549.229 | 2.597 | 0.108 |
| congruency:hemisphere | 0.710 | 0.710 | 1 | 549.221 | 0.972 | 0.325 |
| group:perspective:congruency | 0.687 | 0.687 | 1 | 549.543 | 0.939 | 0.333 |
| group:perspective:hemisphere | 0.017 | 0.017 | 1 | 549.229 | 0.023 | 0.880 |
| group:congruency:hemisphere | 0.134 | 0.134 | 1 | 549.221 | 0.183 | 0.669 |
| perspective:congruency:  hemisphere | 0.074 | 0.074 | 1 | 549.221 | 0.102 | 0.750 |
| group:perspective:congruency:  hemisphere | 4.668 | 4.668 | 1 | 549.221 | 6.387 | 0.012 |

## Table S11. Sensitivity analysis: P200 mean amplitude after exclusion of within-participant conditions with < 15 trials

|  | Sum Sq | Mean Sq | NumDF | DenDF | F value | p |
| --- | --- | --- | --- | --- | --- | --- |
| group | 0.058 | 0.058 | 1 | 78.969 | 0.103 | 0.749 |
| perspective | 0.770 | 0.770 | 1 | 8056.836 | 1.356 | 0.244 |
| congruency | 9.271 | 9.271 | 1 | 8052.641 | 16.327 | 0.000 |
| group:perspective | 1.132 | 1.132 | 1 | 8056.836 | 1.993 | 0.158 |
| group:congruency | 0.778 | 0.778 | 1 | 8052.641 | 1.370 | 0.242 |
| perspective:congruency | 1.524 | 1.524 | 1 | 8052.097 | 2.684 | 0.101 |
| group:perspective:congruency | 0.413 | 0.413 | 1 | 8052.097 | 0.728 | 0.394 |

## Table S12. Sensitivity analysis: P200 50% area latency after exclusion of within-participant conditions with < 15 trials

|  | Sum Sq | Mean Sq | NumDF | DenDF | F value | p |
| --- | --- | --- | --- | --- | --- | --- |
| group | 1.683 | 1.683 | 1 | 80.015 | 2.868 | 0.094 |
| perspective | 0.005 | 0.005 | 1 | 231.016 | 0.009 | 0.924 |
| congruency | 0.004 | 0.004 | 1 | 229.256 | 0.006 | 0.937 |
| group:perspective | 1.819 | 1.819 | 1 | 231.016 | 3.100 | 0.080 |
| group:congruency | 1.001 | 1.001 | 1 | 229.256 | 1.706 | 0.193 |
| perspective:congruency | 0.011 | 0.011 | 1 | 229.151 | 0.018 | 0.893 |
| group:perspective:congruency | 0.100 | 0.100 | 1 | 229.151 | 0.170 | 0.680 |

## Table S13. Sensitivity analysis: P3 mean amplitude after exclusion of within-participant conditions with < 15 trials

|  | Sum Sq | Mean Sq | NumDF | DenDF | F value | p |
| --- | --- | --- | --- | --- | --- | --- |
| group | 0.000 | 0.000 | 1 | 79.101 | 0.000 | 0.995 |
| perspective | 0.677 | 0.677 | 1 | 7991.767 | 0.997 | 0.318 |
| congruency | 58.140 | 58.140 | 1 | 7984.244 | 85.717 | 0.000 |
| group:perspective | 0.340 | 0.340 | 1 | 7991.767 | 0.502 | 0.479 |
| group:congruency | 0.011 | 0.011 | 1 | 7984.244 | 0.016 | 0.900 |
| perspective:congruency | 1.647 | 1.647 | 1 | 7983.334 | 2.428 | 0.119 |
| group:perspective:congruency | 0.027 | 0.027 | 1 | 7983.334 | 0.040 | 0.842 |

## Table S14. Sensitivity analysis: P3 50% area latency after exclusion of within-participant conditions with < 15 trials

|  | Sum Sq | Mean Sq | NumDF | DenDF | F value | p |
| --- | --- | --- | --- | --- | --- | --- |
| group | 0.617 | 0.617 | 1 | 79.195 | 0.921 | 0.340 |
| perspective | 3.985 | 3.985 | 1 | 230.007 | 5.949 | 0.015 |
| congruency | 0.898 | 0.898 | 1 | 227.997 | 1.341 | 0.248 |
| group:perspective | 0.120 | 0.120 | 1 | 230.007 | 0.180 | 0.672 |
| group:congruency | 1.459 | 1.459 | 1 | 227.997 | 2.178 | 0.141 |
| perspective:congruency | 0.038 | 0.038 | 1 | 227.934 | 0.056 | 0.813 |
| group:perspective:congruency | 1.820 | 1.820 | 1 | 227.934 | 2.716 | 0.101 |

## Table S15. Sensitivity analysis: LFSW mean amplitude after exclusion of within-participant conditions with < 15 trials

|  | Sum Sq | Mean Sq | NumDF | DenDF | F value | p |
| --- | --- | --- | --- | --- | --- | --- |
| group | 0.398 | 0.398 | 1 | 79.263 | 0.481 | 0.490 |
| perspective | 11.156 | 11.156 | 1 | 16066.242 | 13.475 | 0.000 |
| congruency | 24.240 | 24.240 | 1 | 16048.976 | 29.279 | 0.000 |
| hemisphere | 190.278 | 190.278 | 1 | 16041.306 | 229.833 | 0.000 |
| group:perspective | 7.110 | 7.110 | 1 | 16066.242 | 8.588 | 0.003 |
| group:congruency | 2.366 | 2.366 | 1 | 16048.976 | 2.858 | 0.091 |
| perspective:congruency | 0.961 | 0.961 | 1 | 16046.756 | 1.161 | 0.281 |
| group:hemisphere | 3.248 | 3.248 | 1 | 16041.306 | 3.923 | 0.048 |
| perspective:hemisphere | 1.489 | 1.489 | 1 | 16041.306 | 1.798 | 0.180 |
| congruency:hemisphere | 0.145 | 0.145 | 1 | 16041.306 | 0.175 | 0.676 |
| group:perspective:  congruency | 2.986 | 2.986 | 1 | 16046.756 | 3.607 | 0.058 |
| group:perspective:  hemisphere | 1.274 | 1.274 | 1 | 16041.306 | 1.538 | 0.215 |
| group:congruency:  hemisphere | 0.077 | 0.077 | 1 | 16041.306 | 0.093 | 0.760 |
| perspective:congruency:  hemisphere | 0.961 | 0.961 | 1 | 16041.306 | 1.161 | 0.281 |
| group:perspective:  congruency:hemisphere | 0.159 | 0.159 | 1 | 16041.306 | 0.192 | 0.661 |

## Table S16. Sensitivity analysis: LFSW 50% area latency after exclusion of within-participant conditions with < 15 trials

|  | Sum Sq | Mean Sq | NumDF | DenDF | F value | p |
| --- | --- | --- | --- | --- | --- | --- |
| group | 0.010 | 0.010 | 1 | 79.175 | 0.014 | 0.908 |
| perspective | 2.570 | 2.570 | 1 | 539.491 | 3.540 | 0.060 |
| congruency | 10.350 | 10.350 | 1 | 535.374 | 14.259 | 0.000 |
| hemisphere | 1.455 | 1.455 | 1 | 533.601 | 2.004 | 0.157 |
| group:perspective | 0.001 | 0.001 | 1 | 539.491 | 0.001 | 0.973 |
| group:congruency | 0.152 | 0.152 | 1 | 535.374 | 0.210 | 0.647 |
| perspective:congruency | 0.766 | 0.766 | 1 | 535.258 | 1.055 | 0.305 |
| group:hemisphere | 0.063 | 0.063 | 1 | 533.601 | 0.087 | 0.768 |
| perspective:hemisphere | 1.271 | 1.271 | 1 | 533.601 | 1.751 | 0.186 |
| congruency:hemisphere | 0.732 | 0.732 | 1 | 533.595 | 1.009 | 0.316 |
| group:perspective:congruency | 1.355 | 1.355 | 1 | 535.258 | 1.867 | 0.172 |
| group:perspective:hemisphere | 0.011 | 0.011 | 1 | 533.601 | 0.015 | 0.901 |
| group:congruency:hemisphere | 0.432 | 0.432 | 1 | 533.595 | 0.595 | 0.441 |
| perspective:congruency:  hemisphere | 0.085 | 0.085 | 1 | 533.595 | 0.117 | 0.732 |
| group:perspective:congruency:  hemisphere | 3.601 | 3.601 | 1 | 533.595 | 4.960 | 0.026 |

## Table S17. Sensitivity analysis: LFSW amplitude after exclusion of n=7 participants fulfilling ICD-10 ADHD criteria (FBB-ADHD)

|  | Sum Sq | Mean Sq | NumDF | DenDF | F value | p |
| --- | --- | --- | --- | --- | --- | --- |
| group | 0.207 | 0.207 | 1 | 72.112 | 0.253 | 0.616 |
| perspective | 9.184 | 9.184 | 1 | 15075.809 | 11.206 | 0.001 |
| congruency | 24.065 | 24.065 | 1 | 15074.785 | 29.365 | 0.000 |
| hemisphere | 184.259 | 184.259 | 1 | 15070.109 | 224.836 | 0.000 |
| group:perspective | 5.455 | 5.455 | 1 | 15075.809 | 6.656 | 0.010 |
| group:congruency | 2.433 | 2.433 | 1 | 15074.785 | 2.968 | 0.085 |
| perspective:congruency | 1.502 | 1.502 | 1 | 15072.594 | 1.832 | 0.176 |
| group:hemisphere | 2.925 | 2.925 | 1 | 15070.109 | 3.569 | 0.059 |
| perspective:hemisphere | 1.678 | 1.678 | 1 | 15070.109 | 2.048 | 0.152 |
| congruency:hemisphere | 0.092 | 0.092 | 1 | 15070.109 | 0.113 | 0.737 |
| group:perspective:  congruency | 2.320 | 2.320 | 1 | 15072.594 | 2.831 | 0.092 |
| group:perspective:  hemisphere | 1.099 | 1.099 | 1 | 15070.109 | 1.341 | 0.247 |
| group:congruency:  hemisphere | 0.107 | 0.107 | 1 | 15070.109 | 0.130 | 0.718 |
| perspective:congruency:  hemisphere | 1.075 | 1.075 | 1 | 15070.109 | 1.312 | 0.252 |
| group:perspective:  congruency:hemisphere | 0.215 | 0.215 | 1 | 15070.109 | 0.262 | 0.609 |

# **Pupillary responses**

## Table S18. SEPR as predicted by group and task conditions (n=55).

|  | Sum Sq | Mean Sq | NumDF | DenDF | F value | p |
| --- | --- | --- | --- | --- | --- | --- |
| group | 0.697 | 0.697 | 1 | 51.577 | 1.027 | 0.316 |
| congruency | 7.949 | 7.949 | 1 | 15147.492 | 11.703 | 0.001 |
| perspective | 1.238 | 1.238 | 1 | 15147.064 | 1.822 | 0.177 |
| time window | 2932.232 | 1466.116 | 2 | 15144.734 | 2158.357 | 0.000 |
| age | 0.547 | 0.547 | 1 | 51.746 | 0.805 | 0.374 |
| group:congruency | 0.416 | 0.416 | 1 | 15147.271 | 0.612 | 0.434 |
| group:perspective | 0.067 | 0.067 | 1 | 15146.909 | 0.099 | 0.753 |
| congruency:perspective | 0.783 | 0.783 | 1 | 15145.508 | 1.152 | 0.283 |
| group:time window | 49.881 | 24.941 | 2 | 15144.735 | 36.717 | 0.000 |
| congruency:time window | 5.493 | 2.746 | 2 | 15143.643 | 4.043 | 0.018 |
| perspective:time window | 3.701 | 1.851 | 2 | 15143.701 | 2.724 | 0.066 |
| group:congruency:  perspective | 0.001 | 0.001 | 1 | 15145.497 | 0.001 | 0.971 |
| group:congruency:  time window | 4.063 | 2.031 | 2 | 15143.643 | 2.991 | 0.050 |
| group:perspective:  time window | 0.472 | 0.236 | 2 | 15143.701 | 0.347 | 0.707 |
| congruency:perspective:  time window | 1.065 | 0.532 | 2 | 15143.636 | 0.784 | 0.457 |
| group:congruency:  perspective:time window | 0.143 | 0.072 | 2 | 15143.633 | 0.106 | 0.900 |

## Table S19. Non-residualized pupil response as predicted by group, task conditions and pupil constriction covariates.

|  | Sum Sq | Mean Sq | NumDF | DenDF | F value | p |
| --- | --- | --- | --- | --- | --- | --- |
| group | 0.095 | 0.095 | 1 | 51.436 | 0.433 | 0.514 |
| congruency | 2.858 | 2.858 | 1 | 15145.096 | 13.048 | 0.000 |
| perspective | 0.441 | 0.441 | 1 | 15144.561 | 2.015 | 0.156 |
| time window | 953.147 | 476.574 | 2 | 15142.400 | 2175.923 | 0.000 |
| age | 0.328 | 0.328 | 1 | 51.639 | 1.498 | 0.227 |
| constriction amplitude | 3106.847 | 3106.847 | 1 | 12666.971 | 14185.130 | 0.000 |
| constriction latency | 70.555 | 70.555 | 1 | 14887.079 | 322.136 | 0.000 |
| group:congruency | 0.107 | 0.107 | 1 | 15144.922 | 0.488 | 0.485 |
| group:perspective | 0.051 | 0.051 | 1 | 15144.795 | 0.231 | 0.631 |
| congruency:perspective | 0.164 | 0.164 | 1 | 15143.507 | 0.748 | 0.387 |
| group:time window | 16.254 | 8.127 | 2 | 15142.405 | 37.105 | 0.000 |
| congruency:time window | 1.811 | 0.906 | 2 | 15141.482 | 4.134 | 0.016 |
| perspective:time window | 1.201 | 0.601 | 2 | 15141.529 | 2.742 | 0.064 |
| group:congruency:  perspective | 0.019 | 0.019 | 1 | 15143.398 | 0.088 | 0.767 |
| group:congruency:  time window | 1.326 | 0.663 | 2 | 15141.476 | 3.028 | 0.048 |
| group:perspective:  time window | 0.156 | 0.078 | 2 | 15141.528 | 0.355 | 0.701 |
| congruency:perspective  :time window | 0.342 | 0.171 | 2 | 15141.472 | 0.780 | 0.458 |
| group:congruency:  perspective:time window | 0.047 | 0.024 | 2 | 15141.470 | 0.107 | 0.898 |

## Table S20. SEPR as predicted by group, task conditions and additional covariate of gaze deviation from the center of the screen.

|  | Sum Sq | Mean Sq | NumDF | DenDF | F value | p |
| --- | --- | --- | --- | --- | --- | --- |
| group | 0.746 | 0.746 | 1 | 51.404 | 1.117 | 0.296 |
| congruency | 8.269 | 8.269 | 1 | 14945.559 | 12.382 | 0.000 |
| perspective | 1.338 | 1.338 | 1 | 14945.289 | 2.003 | 0.157 |
| time window | 2853.681 | 1426.841 | 2 | 14943.491 | 2136.686 | 0.000 |
| age | 0.413 | 0.413 | 1 | 51.489 | 0.619 | 0.435 |
| gaze deviation | 0.019 | 0.019 | 1 | 14683.666 | 0.029 | 0.866 |
| group:congruency | 0.516 | 0.516 | 1 | 14943.968 | 0.773 | 0.379 |
| group:perspective | 0.207 | 0.207 | 1 | 14943.532 | 0.310 | 0.578 |
| congruency:perspective | 0.988 | 0.988 | 1 | 14942.363 | 1.479 | 0.224 |
| group:time window | 57.623 | 28.811 | 2 | 14942.526 | 43.145 | 0.000 |
| congruency:time window | 5.430 | 2.715 | 2 | 14940.700 | 4.066 | 0.017 |
| perspective:time window | 3.756 | 1.878 | 2 | 14940.653 | 2.812 | 0.060 |
| group:congruency:  perspective | 0.010 | 0.010 | 1 | 14942.315 | 0.015 | 0.904 |
| group:congruency:time window | 3.419 | 1.710 | 2 | 14940.490 | 2.560 | 0.077 |
| group:perspective:  time window | 0.501 | 0.251 | 2 | 14940.595 | 0.375 | 0.687 |
| congruency:perspective:  time window | 0.922 | 0.461 | 2 | 14940.417 | 0.690 | 0.502 |
| group:congruency:  perspective:time window | 0.156 | 0.078 | 2 | 14940.418 | 0.117 | 0.890 |

# **Associative analyses**

## Table S21. Group differences in LFSW amplitude with additional predictor of SEPR (TW-3) in the pupillometry subsample.

|  | Sum Sq | Mean Sq | NumDF | DenDF | F value | p |
| --- | --- | --- | --- | --- | --- | --- |
| group | 0.167 | 0.167 | 1 | 57.561 | 0.204 | 0.653 |
| perspective | 16.854 | 16.854 | 1 | 11008.035 | 20.623 | 0.000 |
| congruency | 15.863 | 15.863 | 1 | 10982.354 | 19.410 | 0.000 |
| hemisphere | 118.222 | 118.222 | 1 | 10959.700 | 144.657 | 0.000 |
| SEPR-TW3 | 0.009 | 0.009 | 1 | 1114.301 | 0.011 | 0.917 |
| group:perspective | 5.789 | 5.789 | 1 | 11008.035 | 7.083 | 0.008 |
| group:congruency | 5.137 | 5.137 | 1 | 10982.354 | 6.286 | 0.012 |
| perspective:congruency | 0.946 | 0.946 | 1 | 10965.554 | 1.158 | 0.282 |
| group:hemisphere | 2.652 | 2.652 | 1 | 10959.700 | 3.245 | 0.072 |
| perspective:hemisphere | 2.088 | 2.088 | 1 | 10959.700 | 2.555 | 0.110 |
| congruency:hemisphere | 0.040 | 0.040 | 1 | 10959.700 | 0.049 | 0.825 |
| group:SEPR-TW3 | 0.022 | 0.022 | 1 | 1114.301 | 0.026 | 0.871 |
| group:perspective:  congruency | 2.447 | 2.447 | 1 | 10965.554 | 2.994 | 0.084 |
| group:perspective:  hemisphere | 0.608 | 0.608 | 1 | 10959.700 | 0.744 | 0.388 |
| group:congruency:  hemisphere | 0.122 | 0.122 | 1 | 10959.700 | 0.150 | 0.699 |
| perspective:congruency:  hemisphere | 1.329 | 1.329 | 1 | 10959.700 | 1.626 | 0.202 |
| group:perspective:  congruency:hemisphere | 0.014 | 0.014 | 1 | 10959.700 | 0.017 | 0.895 |

# **Additional information**

## Appendix S1. Simulation-based power analyses

To evaluate the sensitivity of the study design to higher-order interaction effects, we conducted Monte Carlo simulations with 500 repetitions based on the observed number of participants and the realized number of valid trials per participant after preprocessing. Simulations were performed using mixed-effects models matching the structure of the primary analyses, while outcome values were generated under predefined standardized effect sizes. Variance components were specified to yield a conservative degree of within-participant clustering (intraclass correlation coefficient, ICC = 0.30).

For trial-wise analyses in the full sample, simulations indicated adequate sensitivity for moderate three-way interaction effects between group, perspective, and congruency. Specifically, a standardized interaction effect of β = 0.20 was detectable with approximately 80% power (79.6%, 95% CI [75.80, 83.05]), whereas power declined for smaller effects. Two-way interaction effects showed higher sensitivity, with effect sizes of β = 0.15 detectable with over 80% power (group x perspective interaction effect: 86.00%, 95% CI [82.65, 88.92]).

Sensitivity was reduced in the pupillometry subsample due to the smaller number of participants. In the subsample, two-way interaction effects reached approximately 80% power at effect sizes around β = 0.18 (81.80%, 95% CI [78.13, 85.09]), while three-way interaction effects required somewhat larger effect sizes (β ≈ 0.27) to achieve comparable sensitivity (82.40%, 95% CI [78.77, 85.64]). Four-way interaction effects with time window were not the primary focus of the analyses and required larger effect sizes. The simulations indicate that the present study was sufficiently sensitive to detect moderate higher-order interaction effects, while very small effects cannot be ruled out, particularly in the pupillometry subsample.
